# Improving the Impact Resistance and Post-Impact Tensile Fatigue Damage Tolerance of Carbon Fiber Reinforced Epoxy Composites by Embedding the Carbon Nanoparticles in Matrix

**DOI:** 10.3390/polym16243589

**Published:** 2024-12-22

**Authors:** Yi-Ming Jen, Yu-Jen Chen, Tzung-Han Yu

**Affiliations:** Department of Mechanical and Mechatronic Engineering, National Taiwan Ocean University, Keelung 202301, Taiwan; 11072005@email.ntou.edu.tw (Y.-J.C.); 11172010@email.ntou.edu.tw (T.-H.Y.)

**Keywords:** carbon fiber reinforced polymers (CFRP), multiwalled carbon nanotube (MWCNT), graphene nanoplatelet (GNP), low-velocity impact, post-impact static tensile strength, post-impact tensile fatigue strength, damage area growth rate

## Abstract

The effect of dispersing multiwalled carbon nanotubes (MWCNTs) and graphene nanoplatelets (GNPs) in the matrix on the low-velocity impact resistance and post-impact residual tensile strength of the carbon fiber reinforced epoxy composite laminates has been experimentally analyzed in this study. The composite specimens with the matrix reinforced by different nanoparticle types and various nanoparticle concentrations (0.1, 0.3, and 0.5 wt.%) were prepared and impacted. The post-impact tensile quasi-static and fatigue tests were performed on the specimens with different configurations to study the influence of aforementioned factors on the impact resistance and damage tolerance. Experimental results show that adding nanoparticles in the matrix increases the maximum impact force, reduces the damage area, and alleviates the dent depth of the laminates remarkedly. Moreover, the improvement in these impact resistances increases with the applied nanoparticle concentrations. The nano-modified composite laminates present higher post-impact static strength and longer fatigue life than the specimens with a neat epoxy matrix. Furthermore, both the post-impact static tensile strength and fatigue life increase with the applied nanoparticle concentrations. The damage areas measured using infrared thermography were found to increase linearly with the applied fatigue cycles for all the studied specimens with various configurations. The damage area growth rates of nano-modified composite laminates decrease significantly as the applied nanoparticle concentrations increase. The MWCNTs present better performance than GNPs in improving post-impact static strength and extending the residual fatigue life, however the effect of applied nanoparticle type on the fatigue damage growth rate is slight.

## 1. Introduction

Among various materials used in aerospace, shipbuilding, automobiles, wind power and other industries, fiber-reinforced polymer (FRP) composites have become the alternative to traditional metal materials because of their low density, high specific strength and high specific stiffness [1,2,3]. Due to the special manufacturing process and layered structures, the composite laminates exhibit weak interlaminar strength in the out-of-plane direction. Accordingly, the strength design of composite laminates subjected to transverse impact loading has received much attention for decades. Impact loads can be divided into several types based on the impact velocity [4]. Low-velocity impact (<10 m/s) is most commonly experienced by composite laminates in the application, including the following: falling of hand tools or heavy objects; impact of aircraft fuselage from runway debris; hail or bird strikes; collision of vehicles or ships; etc. Moreover, the barely visible impact damage (BVID) caused by the low-velocity impact reduces the stiffness and strength of the composite laminates significantly and increases the risk of usage after impact [5]. Hence, low-velocity impact resistance and the corresponding post-impact damage tolerance have been widely studied, and some review articles are available for reference [6,7,8,9].

Because matrix cracking, fiber splitting, and delamination are the main failure mechanisms observed in the composite laminates subjected to the impact loads, enhancing matrix toughness has been considered an effective method to improve impact strength. Applying matrix materials with high fracture toughness is a straightforward solution to improve the impact resistance [6]. Since the fracture toughness of thermoplastic resins is much higher than that of thermoset ones, thermoplastic polymers have been considered good alternatives to thermoset polymers due to their higher fracture toughness. However, the shortcomings of high process costs and low stiffness limit the application of thermoplastic composites

In the past two decades, many studies have been devoted to applying carbon nanofillers in fiber reinforced polymer composites to enhance various mechanical properties. These efforts have yielded excellent results and can be surveyed in some review papers [10,11,12,13]. Among these nano-enhancement techniques, the matrix toughening method [14,15] has been applied extensively to enhance the interlaminar fracture toughness of the laminates. Among the various nanoparticle candidates, carbon nanofillers have been employed frequently to modify the mechanical properties of polymer materials [16]. In the past two decades, many efforts have been made to disperse carbon nanoparticles uniformly in the matrix to improve the fracture toughness of composites, thereby increasing the impact resistance and damage tolerance [15,17,18]. These carbon nanoparticles can be divided into several categories according to their nano-dimensionalities. The carbon nanotube (CNT) family and graphene-based materials are the most frequently utilized one-dimensional (1D) and two-dimensional (2D) carbon nano-reinforcements to enhance the impact strength of composite materials. The advantages of CNTs on the improvement of impact and post-impact strengths for composites have been reported in several studies. In 2010, Kostopoulos et al. [19] investigated the effect of adding multi-walled carbon nanotubes (MWCNTs) in the matrix on the impact and post-impact behaviors of carbon fiber-reinforced polymer (CFRP) composite laminates. Experimental results verified that adding MWCNTs improved the impact resistance and post-impact compressive static/fatigue strengths of the laminate specimens. The glass fiber-reinforced polymer (GFRP) laminates with different contents of CNTs were prepared by Venkatanarayanan and Stanley [20] to study the medium-velocity impact characteristics. The results revealed that adding CNTs in the matrix improved the ability of absorbing impact energy of studied composites. Tehran et al. [21] studied the influence of mixing MWCNTs in the matrix on the impact resistance and damage of the CFRP composite laminates in 2013. The addition of multi-walled carbon nanotubes was found to be beneficial to the impact resistance, but Young’s modulus and strength were almost unaffected as the tensile strain increased.

In 2014, Xu et al. [22] employed solvent casting technology to prepare pure polyetherketone (PEK-C) film and MWCNTs hybrid PEK-C mixed (MWCNTs/PEK-C) film with different MWCNT contents. The neat and nano-hybrid films were used as the interleaves for carbon fiber/bismaleimide (BMI) composite laminates. The experimental results showed that the impact damage area of laminates with PEK-C and MWCNTs/PEK-C film tended to decrease compared with the neat laminates. Compared with baseline composite laminates, the post-impact compressive strength of interleaved composite laminates was improved remarkably. The following year, Rahman et al. [23] mixed the prepregs with 1 wt.% oxidized carbon nanofibers (O-CNFs) to prepare the CFRP composite laminates. Low-velocity impact tests were conducted on the laminate specimens at various energy levels. The experimental results showed that the nanofibers were evenly dispersed in the epoxy resin and there was good adhesion between the nanofibers and the epoxy resin. The interfacial bonding between the fiber and matrix was also improved. The improvement in the impact damage tolerance of CFRP laminates with the matrix modified by the MWCNTs was reported by Rawat and Singh [24] in the same year. It showed that the addition of MWCNTs could enhance the impact damage tolerance of studied CFRP composites and 0.25 wt.% was the optimal concentration.

The impact strength of CNT- and nanoclay-modified CFRP composites was investigated by Mahdi et al. [25] in 2017. The results demonstrated that adding CNTs or nanoclays alone enhanced the impact resistance and slightly reduced the impact damage area. However, the application of hybrid nanoparticles in the matrix improved the impact properties of CFRP composites significantly. In 2018, Kara et al. [26] studied the low temperature effect on the low-velocity impact behavior of MWCNT-modified CFRP composite tubes. The experimental results showed that the damage of composite pipes increased as the temperature decreased. Adding nanoparticles in the matrix increased the impact force and reduced the damage area of the composite tubes substantially.

In 2019, Ismail et al. [27] studied the effect of adding MWCNTs to the matrix on the impact behavior and post-impact compressive strength of flax/carbon fiber composites and flax/glass fiber composites. The results showed that the flax/glass fiber composites presented better impact properties than the flax/carbon fiber composites, and the flax/carbon fiber composites experienced more serious damage. In the same year, Nor et al. [28] also explored the influence of dispersing MWCNTs in the matrix on the low-velocity impact response and post-impact compression properties of natural fiber-mixed composites. The experimental results showed that dispersing 0.5 wt.% MWCNTs in the matrix reduced the absorbed energy of the studied composite by 9.21% and increased the maximum impact force by 36.23%. It was found that the impact damage of the studied composites reduced when the MWCNTs were added in the matrix. Furthermore, adding MWCNTs also increased the post-impact compressive strength of the composite laminates by 23.67%.

In 2019, Ranjbar and Feli [29] studied the effects of adding MWCNTs with different concentrations on the low-velocity impact response of pure epoxy resin and epoxy resin/glass fiber composite beams. The experimental results revealed that adding 0.34 wt.% MWCNTs improved the low-velocity impact strength and compressive property after impact of GFRP composite beams significantly. In 2020, Demircan et al. [30] mixed MWCNTs in the matrix to prepare nano-modified glass fiber/epoxy composites for the impact and post-impact compressive tests. The experimental results showed that the post-impact stiffness and strength of MWCNT-reinforced composites increased by 26% and 17%, respectively. Compared to the baseline composites, the impact strength was found to increase by 17%. The number of cracks in the MWCNT-modified specimen was less and the crack length was shorter than those of the specimen with a neat matrix. Xin et al. [31] also studied the effect of randomly oriented MWCNT interleave on the low-velocity impact behavior of CFRP laminates in the same year. The results demonstrated that the delamination area of the MWCNT-modified laminate reduced by 11–39% compared to the baseline composites.

In 2021, Yıldırım et al. [32] studied the low-velocity impact performance of three-dimensional woven glass fiber/epoxy resin composites at low-temperatures. Experimental results showed that three-dimensional composites can absorb higher impact energy. Moreover, the MWCNTs and silica were applied in the matrix to improve the impact performance and damage characteristics of the studied composites. The impact resistance of the nano-modified specimens was found to increase by 30%. The improvement in impact strength was obvious at room temperature but decreased with the temperature.

In 2022, Praneeth et al. [33] evaluated the mechanical properties of CFRP composites with various contents of MWCNTs. The results found that the impact strength of MWCNT-modified CFRP composites increased by 40% compared to the baseline composites. It was also found that fiber fracture and pullout were the main enhancement mechanisms. Badawy and Khashaba reported that adding CNTs in the epoxy matrix increased the impact force and absorbed energy of Kevlar and glass fiber composites by 95% and 11%, respectively [34]. In the same year, Sergi [35] et al. studied the toughening effect of adding MWCNTs in the matrix on the impact and post-impact response of CFRP laminates with different stacking sequences. Experimental results showed that quasi-isotropic laminates showed a higher impact damage tolerance than the orthogonal ones. In addition, it was found that CNTs displayed a significant toughening effect on quasi-isotropic laminates at all considered temperatures, and the residual bending strength was increased by 10%. The enhancement mechanism of damage tolerance was attributed to the bridging and pullout effects provided by CNTs.

Compared with the studies about the contributions of 1D carbon nanofillers to the impact strength of FRP composites, few studies on the improvement of impact resistance using graphene-based nano-reinforcement are available. In 2018, Amooyi Dizaji et al. [36] conducted impact response experiments on 3D woven glass fiber-braided composite cores materials. The silica and graphene were mixed in the matrix to improve the impact energy absorption behavior. The experimental results also showed that plain woven composite specimens presented higher impact resistance than the 3D glass fiber-braided composite materials. Domun et al. [37] studied the effect of adding nano-reinforcements to glass fiber-reinforced polymer composites on ballistic impact response in 2019. In the study, four types of nano-reinforcements were employed to improve the impact strength of GFRPs, i.e., graphene nanoplatelets (GNPs), CNTs, boron nitride nanotubes (BNNS)/CNTs, and BNNS/GNPs. The experimental results showed that composites with nano-reinforcements presented better impact performance than the ones with the neat matrix. Moreover, the specimens with BNNS/GNPs displayed the highest absorbed energy, which was 16.8% higher than the composite laminates without nano-reinforcements. Elmarakbi et al. [38] experimentally evaluated the effect of adding GNPs of two different sizes (5 and 30) in the matrix on the impact behavior of composite laminates. The experimental results showed that using GNPs with smaller size had a negative effect on the maximum impact force and absorbed energy of the composites, while adding GNPs with larger size increased the absorbed energy markedly. Next year, Alsaadi et al. [39] found that adding graphene in the matrix could improve the impact strength of the hybrid composites with carbon/aramid/carbon and aramid/carbon/aramid layered architectures by 2% and 8%, respectively.

Surveying the studies concerning the achievement of applying the carbon nanofillers to improve the impact strength of FRPs indicates that the statically compressive tests are the most frequently adopted method to evaluate the post-impact damage tolerance. Although the indentation, delamination and local buckling caused by the impact loading reduce the resistance of FRPs to the compressive loading, it is equally important to assess the residual tensile strength after impact for the fiber splitting, fiber breakage and matrix cracking are common damages caused by impact. In addition, as an innovative engineering material, the nano-modified FRPs are frequently subjected to the fluctuating loading in service life. Accordingly, the knowledge regarding the impact resistance and post-impact tensile fatigue characteristics of the FRPs with the matrix toughened by the nanoparticles is needed urgently. The goal of this study is to experimentally investigate the effect of adding carbon nanoparticles in the matrix on the low-velocity impact resistance and post-impact tensile static/fatigue strengths of the carbon fiber-reinforced epoxy composite laminates. The MWCNT-modified and GNP-modified FRP specimens with various nanoparticle concentrations were prepared for the impact and post-impact tests to study the effects of nanoparticle type and concentration on the impact resistance and damage tolerance under tensile loading.

## 2. Materials and Methods

### 2.1. Materials and Specimen Preparation

The 12K unidirectional TR50S carbon fabrics (Mitsubishi Chemical Carbon Fiber and Composites, Inc., Yokkaichi, Japan) were applied as the reinforcements of the studied composite laminates. The fiber areal weight and the thickness of each ply were 222 g/m^2^ and 0.4 mm, respectively. The solvent-type bisphenol A epoxy, fabricated by Yu Po Chemical Co. Ltd., Tainan City, Taiwan with the designation of TTP-3X, was employed as the material of matrix. The MWCNTs and GNPs were applied as the 1D and 2D nano-reinforcements to modify the epoxy matrix in the study. The MWCNTs were supplied by Nanocyl Co. Ltd., Sambreville, Belgium, with the designation of NC7000. The length and diameter of the MWCNT were approximately 1.5 μm and 9.5 nm, respectively. The carbon purity was higher than 90%. The GNPs were manufactured by Xiamen Knano Graphene Technology Co. Ltd., Xiamen, China, with the product identification of KNG-150. The diameter of the employed GNP was approximately 5 μm and the thickness ranged from 5 to 15 nm. The carbon purity was higher than 99.5%.

To prepare the nano-modified epoxy resin, the dried MWCNTs or GNPs with required amount were added in the MEK solvent first and agitated using an ultrasonic homogenizer. After being agitated for 10 min, the suspension was mixed with the surfactant Triton-X405 and blended continuously for another 20 min to obtain the uniform dispersion of carbon nano-particles in the methyl ethyl ketone (MEK) solvent. Next, the suspension was mixed with the solvent-type epoxy resin using a planetary centrifugal mixer for 20 min to obtain the nano-modified epoxy resin.

The nano-modified epoxy resin with controlled nanoparticle concentration was smeared uniformly on the unidirectional fabric with the dimension of 350 mm × 350 mm using a roller brush and a scraper. The impregnated fabric was stored at room temperature for 24 h and then heated at 80 °C in a vacuum dry oven for 40 min to obtain the prepreg. The composite laminate was manufactured by conventional hand lay-up process. Four layers of fabrics with the ply stacking sequence of [0/90/90/0] were hot-pressed in a mold at 150 °C with the pressure rising from 0 to 1500 psi gradually in 5 min followed by the constant pressure of 1500 psi for 20 min. The obtained composite laminate was cut to the impact specimens with required dimensions using a precision diamond saw. The impact specimens with six configurations were prepared by considering the factors of the employed nanoparticle type (MWCNT or GNP) and the concentration of the nanoparticle in the epoxy matrix (0.1, 0.3 and 0.5 wt.%). The specimens with the neat matrix were also prepared for the comparative purpose.

### 2.2. Experimental Methods

All the impact tests for the specimens with different configurations were performed according to ASTM standard D7136 [40] by using a weight-drop tower (Yu-Da Co., Tainan City, Taiwan) at an impact energy level of 12 J. The impact energy was controlled by adjusting the height of the 5.5 kg hemisphere impactor with the diameter of 16 mm

The specimen was clamped by two rigid plates with an open window for impact. The impact force, impactor displacement and velocity were recorded during the impact test. After the impact test, the indentation was measured using a digital dial gage. The thermal image of the impacted specimen was obtained to analyze the damage area using an infrared thermometer (NEC G100EX, Tokyo, Japan). The format of the obtained thermal image was transferred to the binary image type, and the damage boundary edge was judged and determined by selecting a threshold value for the image gradient in processing the binary image. Then, the damage area was obtained by calculating the pixels within the boundary [41,42].

**Figure 1:**
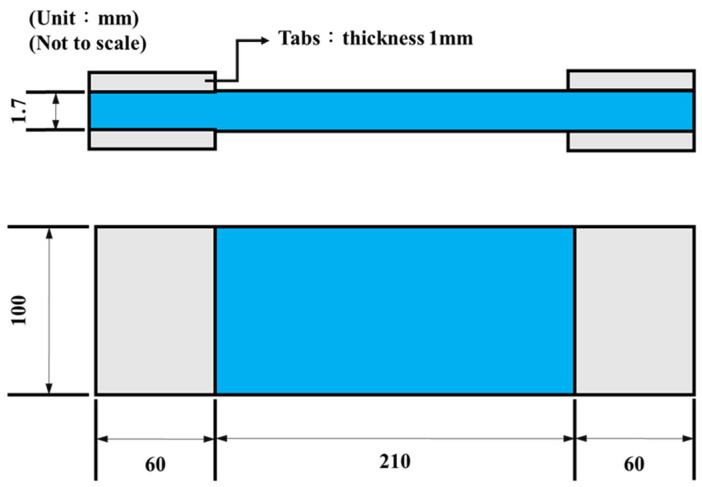
Shape and dimensions of the specimen for the post-impact tensile tests.

After the impact test, the aluminum end-tabs with 1 mm thickness were mounted on the impacted specimen using an epoxy structural adhesive, and the quasi-static tensile test was performed on the impacted specimen using an MTS 810 servo-hydraulic material testing system (MTS Systems Corporation; Eden Prairie, MN, USA). The shape and dimensions of the specimen for the post-impact tensile test are illustrated in Figure 1. The tensile test was conducted by controlling the speed of crosshead at 1.25 mm/min, and both the applied load and displacement of crosshead were recorded for future analysis. The post-impact tensile strength *σ*_TAI_ was defined as the ratio of ultimate applied load to the gross cross-sectional area of the specimen.

Next, the residual fatigue behavior after impact of the specimens was experimentally studied. The impacted specimens were fatigue-tested by load control. The waveform of the applied cyclic load was sinusoidal with the frequency of 3 Hz. Two loading levels, i.e., 85% and 90%, were selected to perform the fatigue tests. The loading level was defined as the ratio of the maximum applied load in a fatigue cycle to the maximum tensile load obtained in the post-impact statically tensile test. The fatigue life *N_f_* is defined as the cycle number corresponding to the specimen separation. The fatigue test was interrupted when the loading cycles exceeded 90,000 cycles. Moreover, the thermographs of the fatigue tested specimen were monitored and recorded at regular cycle intervals using an infrared thermometer. These obtained thermographs were analyzed to determine the damage area using aforementioned image processing method to study the damage evolution in the fatigue test.

The fracture surfaces of the fatigued specimens were observed using a field emission scanning electron microscope (SEM) (Hitachi Regulus-8100, Tokyo, Japan) to examine the reinforcement mechanisms on the impact resistance and post-impact fatigue characteristics.

## 3. Results

### 3.1. Low-Velocity Impact Resistance

**Figure 2:**
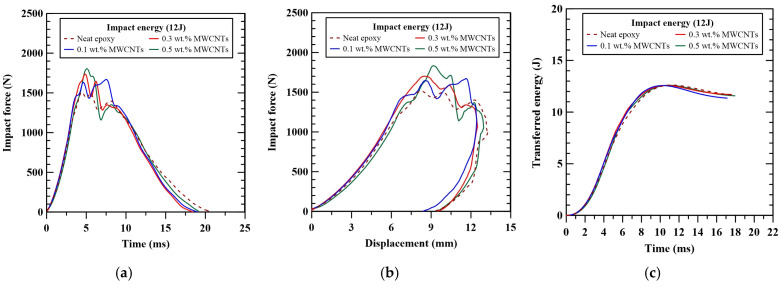
The influence of nanoparticle concentrations on the (**a**) impact force-time, (**b**) impact force-displacement, and (**c**) transferred energy-time curves of the representative MWCNT-modified CFRP laminates.

The experimental data of impact response, such as maximum impact force and absorbed energy, obtained from the impact tests for the studied nano-modified CFRP laminate specimens with various nanoparticle concentrations, are listed in Table 1. Figure 2 shows the influence of nanoparticle concentrations on the impact response of the representative MWCNT-modified CFRP laminates. Figure 2a shows that adding MWCNTs in the matrix of the studied composite laminates increases the maximum impact force and reduces the impact duration. Moreover, the maximum impact force increases with the applied MWCNT concentration. The timing corresponding to the maximum impact load is found to be delayed when the employed nanoparticle concentration increases. Figure 2b demonstrates that all the MWCNT-reinforced laminate specimens with various nanoparticle concentrations present rebound behavior when subjected to impact loading. The nano-modified specimens experience lower deflection than the specimens with a neat epoxy matrix. No obvious differences in the transferred energy behavior are found between the specimens with the matrix modified by various contents of MWCNTs, as depicted in Figure 2c. Only few impact energies were recovered elastically; approximately, 92% of impact energy is absorbed by the laminate specimens.

**Figure 3:**
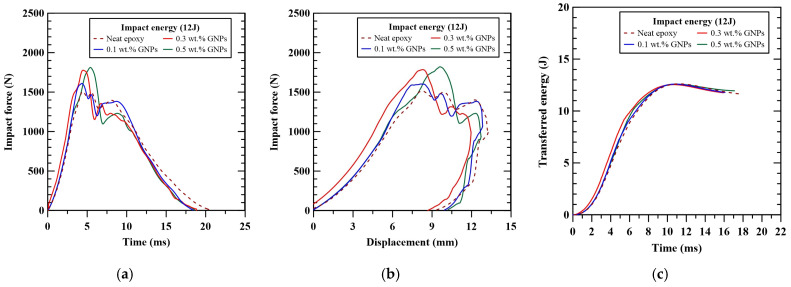
The influence of nanoparticle concentrations on the (**a**) impact force-time, (**b**) impact force-displacement, and (**c**) transferred energy-time curves of the representative GNP-modified CFRP laminates.

The impact response for the representative GNP-modified laminate specimens is shown in Figure 3. Similar to the behavior of MWCNT-modified specimens, the maximum impact force increases with the content of the GNPs, as shown in Figure 3a. A later timing of the peak impact force was found when more nanoparticles were dispersed in the matrix. Furthermore, Figure 3b shows that regardless of the applied nanoparticle content, all the GNP-modified specimens exhibit rebound behavior when subjected to the 12 J impact loading. The addition of GNPs in the matrix can reduce the maximum deflection and permanent deformation of the laminate specimens effectively. The trends in transferred energy for the specimens with the matrix modified by different contents of GNPs are similar, as illustrated in Figure 3c, implying that obvious damage was initiated during the impact tests.

**Figure 4:**
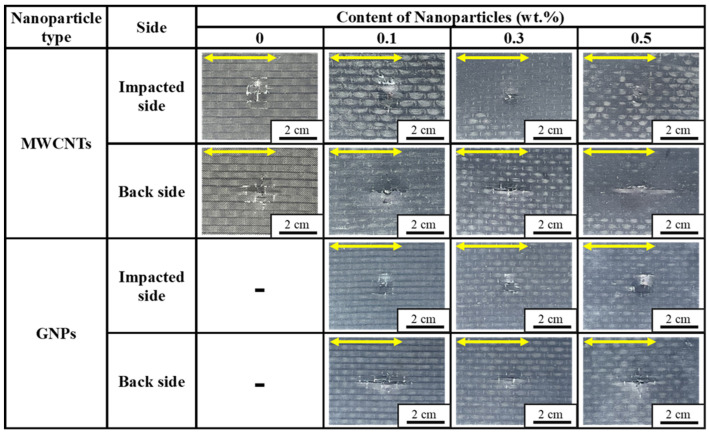
Representative photographs taken from the impacted sides and back sides of the studied CFRP specimens with various configurations after impact. The arrows indicate the length directions of the specimens.

Figure 4 shows the representative photographs taken from the impacted sides and back sides of the studied specimens with various configurations after impact. All the photographs of the impacted sides of specimens present circular-shaped indentations, which coincide with the profile of the impactor. The photographs of the back sides of the specimens show that the fiber splitting, fiber fracture, matrix cracks, delamination, and sub-buckling dominate the impact damage. The damage developed along both the longitudinal and transverse directions of the specimen, which corresponded to the fiber directions of last two layers of fabrics (0° and 90°). Furthermore, the damage growing along the longitudinal direction is longer than that propagating along the transverse direction, implying that the matrix strength/toughness and the fiber/matrix interfacial strengths dominate the impact damage.

**Figure 5:**
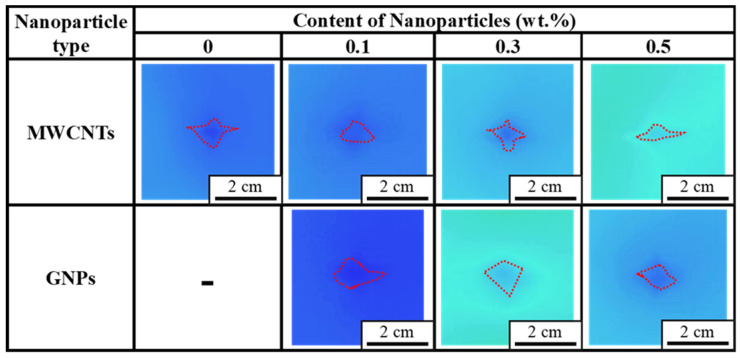
Representative thermographs taken from the back sides of the studied CFRP specimens with various configurations after impact.

For the purpose of quantification, the damages at the back sides of all the impacted specimens were observed using an infrared thermometer and the damage areas were determined by the image processing method. Figure 5 shows the obtained thermographs of the impacted laminated specimens with the matrix modified by various concentrations of nanoparticles. The shapes of damage observed using the infrared thermography are similar to those obtained visually (Figure 4). The damage in the longitudinal direction is greater than that in the transverse direction. The red dashed contour shown in each thermograph of Figure 5 represents the boundary of the damaged regions obtained using the edge finding function of image processing method. The experimental data of damage area observed from the studied impacted specimens with different configurations using infrared thermography have been summarized in Table 1. Similar to Figure 4, the longer damage was observed along the longitudinal direction than that along the transverse direction. Moreover, with the image processing program, the damage area can be calculated for future analysis.

**Figure 6:**
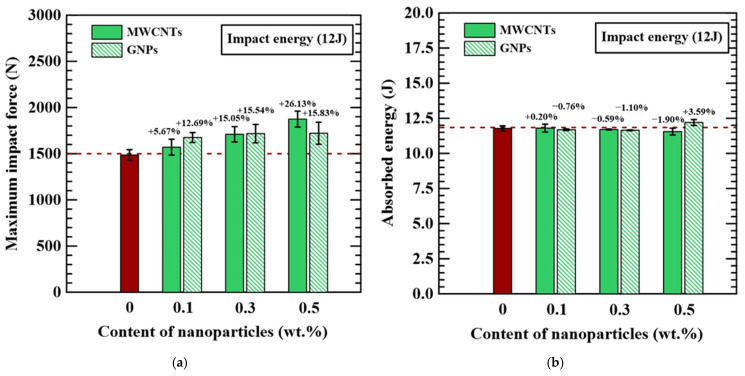

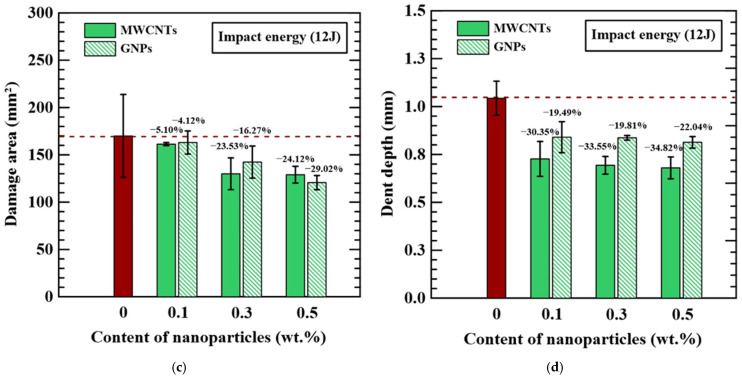
The effects of nanoparticle types and applied concentrations on the (**a**) maximum impact force (**b**) absorbed energy, (**c**) damage area, and (**d**) dent depth of the studied impacted specimens with different configurations. The red column denotes the data of neat CFRP, and the dotted line indicates the average value.

Figure 6a,b summarize the effects of nanoparticle types and applied concentrations on the maximum impact force and absorbed energy of the studied impacted specimens with different configurations, respectively. The experimental results show that adding nanoparticles in the matrix can increase maximum impact force effectively, and the improvement in impact force increases with the applied contents of nanoparticles. Compared to the specimens with a neat epoxy matrix, the maximum impact forces for the specimens with the matrix modified by 0.5 wt.% MWCNTs and GNPs increase by 26% and 16%, respectively. It implies that the application of nanoparticles can enhance the toughness of the polymer matrix and the fiber/matrix interfacial strength, further improving the impact resistance of the CFRP laminate specimens. Since the 1D nanoparticles have better dispersion performance in the polymer matrix than the 2D ones [43], the MWCNT-modified specimens exhibit better impact strength than the GNP-modified ones under the same applied concentration. However, there were only slight effects of nanoparticle types and concentrations on the absorbed energy of the studied specimens. The magnitudes of absorbed energy for the specimens with the matrix modified by various concentrations of nanoparticles are almost identical to those of the specimens with a neat epoxy matrix. Because the application of nanoparticles can increase the impact force and decrease the deflection of the impact response, the absorbed energy of the nano-modified specimen obtained from the area enveloped the force–displacement curve almost remains unchanged compared to the specimens with a neat epoxy matrix.

The damage area and dent depth of impacted CFRP specimen are two physical indicators to evaluate the impact resistance and to assess the tolerance of damaged laminates subjected to post-impact loading. Figure 6c,d show the comparison in damage area measured using the inferred thermography and the dent depth between the specimens with different configurations, respectively. Identical trends of damage area and dent depth were found for the MWCNT-modified specimens and GNP-modified specimens. The more amounts of nanoparticles were applied in the matrix, the less damage area and dent depth were observed in the impacted specimens. Adding 0.5 wt.% MWCNTs and GNPs in the matrix can reduce the damage area of the CFRP laminate specimens by 24% and 29%, respectively. Furthermore, the dent depths of the studied CFRP laminate specimens decrease by 35% and 22% when dispersing 0.5 wt.% MWCNTs and GNPs in the matrix, respectively. The application of nanoparticles can reduce the permanent impacted degeneration by improving the toughness and strength of the polymer matrix effectively. Moreover, the suppressed damage is beneficial to the post-impact stiffness and strength of the specimens when subjected to the subsequent mechanical loading.

### 3.2. Post-Impact Statically Tensile Strength

**Figure 7:**
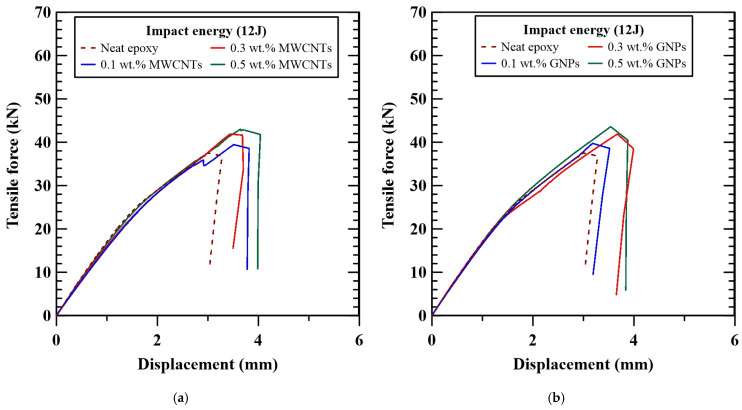
Applied load–displacement curves obtained in the post-impact statically tensile tests for the (**a**) MWCNT-modified CFRP laminates and (**b**) GNP-modified CFRP laminates.

**Figure 8:**
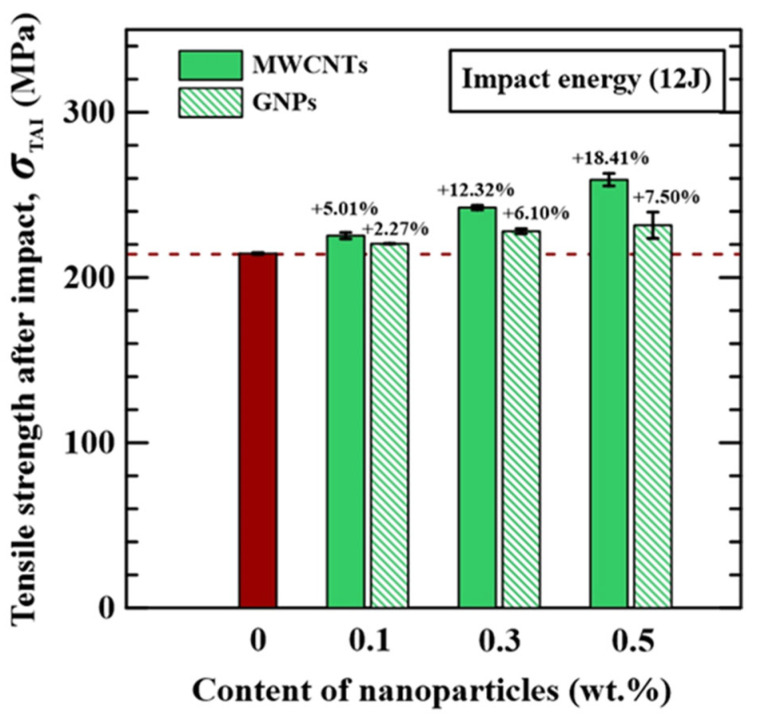
The effects of nanoparticle types and concentrations on the post-impact statically tensile strengths of the nano-modified CFRP laminates. The red column denotes the data of neat CFRP, and the dotted line indicates the average value.

Figure 7a,b show the effect of nanoparticle concentrations on the load–displacement behaviors obtained from the post-impact statically tensile tests for the MWCNT-modified specimens and GNP-modified specimens, respectively. All specimens with different configurations display similar load–displacement behavior. A nonlinear load–displacement relationship was observed before the peak force was reached. At the same time that the load–displacement curve reached the peak point, the fiber fracture occurred and then the force began to decrease rapidly. Moreover, regardless of MWCNT-modified specimens or GNP-modified ones, the effect of nanoparticle concentrations on the tensile stiffness of the studied nano-modified CFRP specimens is slight. Oppositely, the studied CFRP laminate specimens with various nanoparticle concentrations demonstrate different post-impact tensile strengths. The post-impact tensile strengths, *σ*_TAI_, based on the peak values of applied loads in the load–displacement curves, are also listed in Table 1. Figure 8 shows the influence of nanoparticle type and concentration on the post-impact static tensile strengths of the studied MWCNT-modified specimens and GNP-modified specimens, respectively. The residual tensile strengths increase with the applied concentrations of the nanoparticles. The post-impact tensile strengths of the specimens with the matrix modified by 0.5 wt.% MWCNTs and 0.5 wt.% GNPs are 18% and 8% higher than that with a neat epoxy matrix, respectively. The MWCNTs are found to have better improved achievements in post-impact tensile strength than GNPs. The agglomeration of GNPs resulting from the Van der Waals forces between the layered structures weakens the reinforcement performance in toughing the polymer matrix [44], further reducing the post-impact static strength.

### 3.3. Post-Impact Tensile Fatigue Behavior

Table 2 lists the experimental results of post-impact tensile fatigue life for the studied CFRP laminate specimens with various configurations. The obtained fatigue lives for all specimens fatigue tested at 80% load level are longer than 90,000 cycles, while the fatigue lives for the studied specimens with different nano-modification conditions range from 10,000 to 40,000 cycles when tested at 90% level, depending on the applied nanoparticle type and concentration.

**Figure 9:**
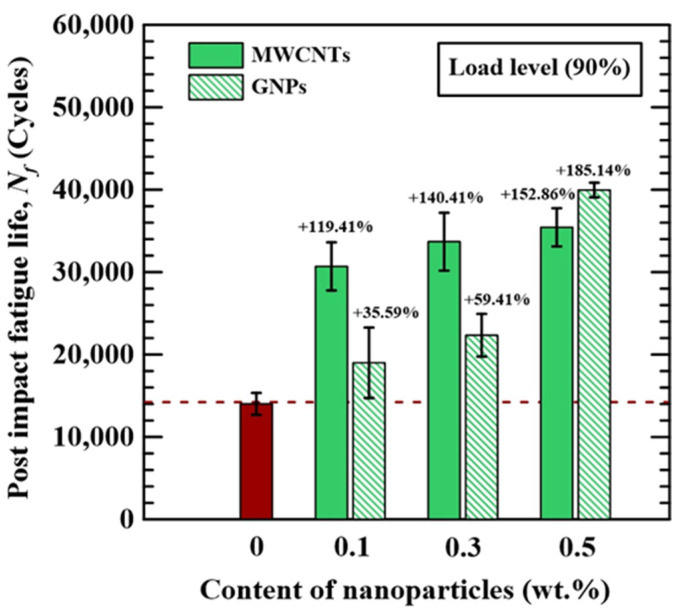
The effects of nanoparticle types and concentrations on the post-impact fatigue lives of the nano-modified CFRP laminates. The red column denotes the data of neat CFRP, and the dotted line indicates the average value.

Figure 9 compares the post-impact fatigue lives tested at 90% load level between the studied CFRP laminate specimens with different nano-modification conditions. It is seen that adding nanoparticles in the polymer matrix extends the post-impact fatigue strength of the bulk CFRP laminates significantly, and the fatigue life increases with the applied nanoparticle concentration. The CFRP laminates with the matrix modified by 0.5 wt.% MWCNTs and 0.5 wt.% GNPs are 1.5 and 1.8 times the post-impact fatigue life of the laminates with a neat epoxy matrix, respectively. Moreover, when the applied nanoparticle concentrations are 0.1 and 0.3 wt.%, the MWCNT-modified specimens present longer lives than the GNP-modified ones. The difference in fatigue life between the MWCNT-modified laminate and GNP-modified CFRP one with the matrix reinforced by 0.5 wt.% nanoparticle concentrations is slight. The reason that the MWCNT-modified CFRP laminate displays better residual fatigue resistance is that the as-impacted damage area of the MWCNT-reinforced specimen is smaller than that of the GNP-modified specimen.

**Figure 10:**
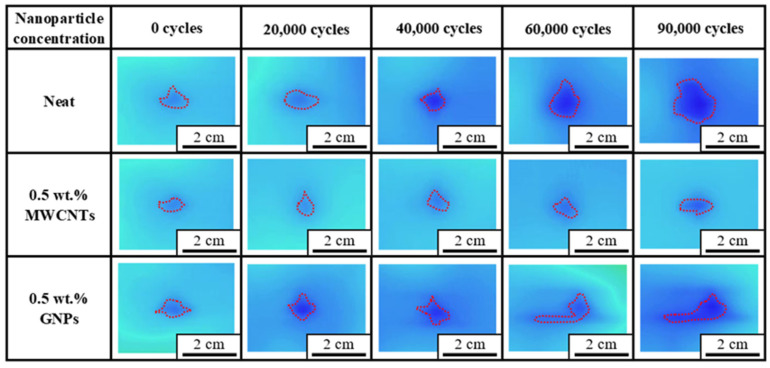
Comparison of damage evolution between the impacted CFRP laminate specimens with a neat epoxy matrix and those with the matrix modified by 0.5 wt.% nanoparticles fatigue, tested at 80% load level.

**Figure 11:**
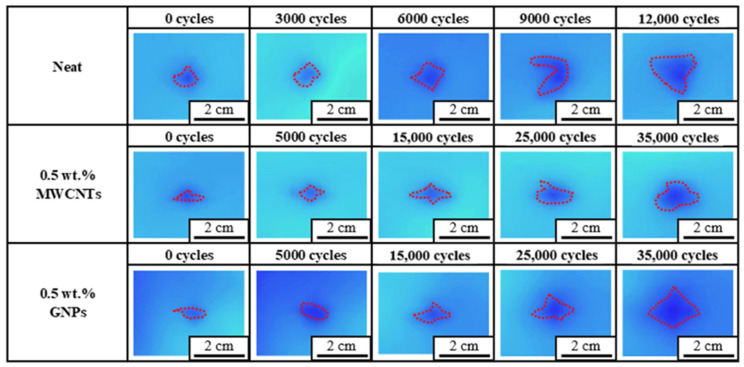
Comparison of damage evolution between the impacted CFRP laminate specimens with a neat epoxy matrix and those with the matrix modified by 0.5 wt.% nanoparticles fatigue tested at 90% load level.

Figure 10 and Figure 11 show the comparison in evolution of the damage area between the impacted CFRP laminate specimens with a neat epoxy matrix and the ones with the matrix modified by 0.5 wt.% nanoparticles fatigue tested at 80% and 90% load levels, respectively. The damage areas with red dashed contours, shown in Figure 10 and Figure 11, were determined at constant intervals of cycles by the infrared thermography. Both figures demonstrate that the damage area increases with applied fatigue cycles, and the observed damage area for the nano-modified specimen is smaller than that for the specimen with a neat epoxy matrix obtained at the same number of fatigue cycles. It implies that the nanoparticles in the matrix retard the damage propagation effectively, further improving the post-impacted fatigue strength of the CFRP laminates.

**Figure 12:**
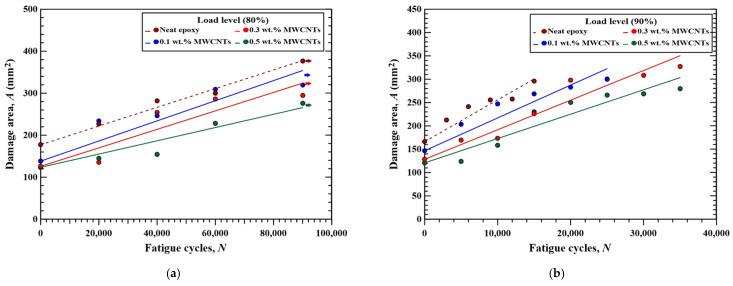
Variations of the measured damage area with the fatigue cycles for the MWCNT-modified CFRP specimens under various concentration conditions, fatigue tested at (**a**) 80% load level and (**b**) 90% load level.

**Figure 13:**
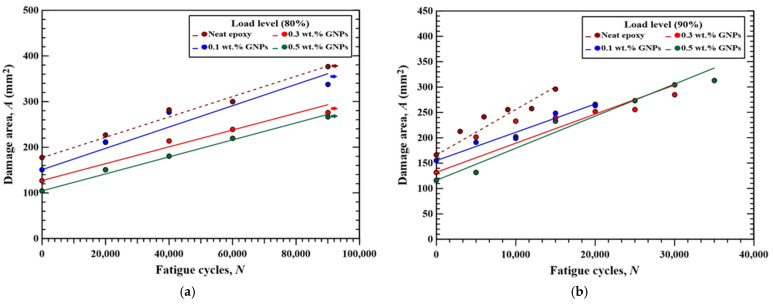
Variations in the measured damage area with the fatigue cycles for the GNP-modified CFRP specimens under various concentration conditions, fatigue tested at (**a**) 80% load level and (**b**) 90% load level.

Figure 12 and Figure 13 show the variations in the measured damage area with the fatigue cycles for the MWCNT-modified CFRP specimens and GNP-modified CFRP specimens under various concentration conditions, respectively. For the specimens modified by specific nanoparticle type and concentration, the trend that the damage area increases with the fatigue cycle is evident. A linearly increasing equation can be used to describe the relationship between the damage area and applied fatigue cycles, which can be expressed as follows:

*A*(*N*) = *A*_0_ + *mN*, (1)

where *A*(*N*) is the damage area at *N*th cycles, and *A*_0_ is the initial damage area measured from the as-impacted specimen before the fatigue test. The fitting results of slope *m* is the predicted damage area growth rate *dA*/*dN*, which is dependent on the load level adopted in the fatigue test, nanoparticle type, and nanoparticle concentration applied in the specimen. Table 3 lists the fitting results of *dA*/*dN* obtained using the proposed model. All the coefficients of determination *R*^2^ of the fitting results for the CFRP specimens with different configurations are higher than 0.83, indicating that the proposed model is appropriate to evaluate the damage area at specific fatigue cycles. The fitting results shown as the straight lines are also depicted in Figure 12 and Figure 13.

**Figure 14:**
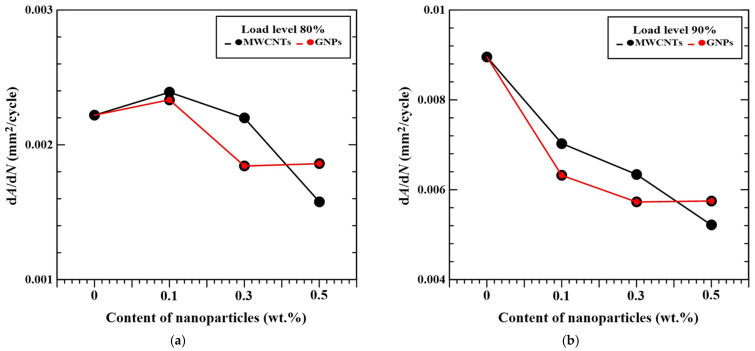
Variation in damage area growth rates with the employed nanoparticle concentrations for the studied nano-modified CFRP laminate specimens, fatigue tested at (**a**) 80% load level and (**b**) 90% load levels.

Figure 14a,b summarize the variation in damage area growth rate with the employed nanoparticle concentrations for the studied nano-modified CFRP laminate specimens fatigue tested at 80% and 90% load levels, respectively. Comparison between Figure 14a,b presents that for the specimens with identical nanoparticle type and concentration, the damage growth rate of the specimen fatigue tested at 90% load level is 2.5 to 4 times higher than that fatigue tested at 80% load level. Furthermore, the specimens with the matrix modified by higher concentrations of nanoparticles have lower damage propagation rate except for the case where the matrix reinforced by 0.5 wt.% GNPs. The addition of 0.5 wt.% MWCNTs in the matrix reduce the damage area growth rate at 80% and 90% load levels by 24% and 47% compared to the ones with a neat epoxy matrix, respectively. The CFRP specimens with 0.3 wt.% GNPs also show 26% and 41% lower damage area growth rate than the control group specimens when fatigue tested at 80% and 90% load levels, respectively. However, owing to the stress concentration effect, the nanoparticle agglomeration caused by the GNPs with high concentrations declines the resistance to the fatigue damage propagation [45].

Figure 14a,b also demonstrate that the effect of applied nanoparticles type in the matrix on the post-impact damage area growth rate is slight. The maximum differences in damage growth rate between the specimens with identical concentration but different nanoparticle type are less than 2.2 × 10^−4^ and 1.1 × 10^−3^ mm^2^/cycle when fatigue tested at 80% and 90% load levels, respectively.

**Figure 15:**
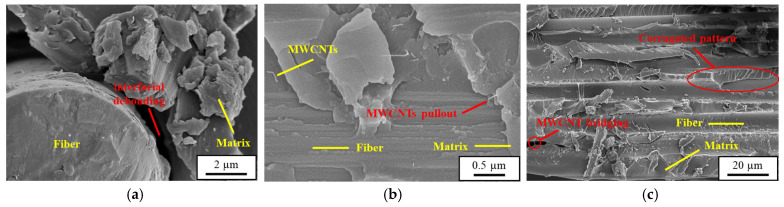
(**a**) Fiber/matrix debonding observed in the SEM image obtained after the post-impact fatigue test for the CFRP specimen with a neat matrix. (**b**) Pullout of MWCNTs. (**c**) MWCNT-bridging effect observed in the SEM images obtained after the post-impact fatigue tests for the MWCNT-modified CFRP specimens.

Figure 15a shows the representative SEM images taken from the fatigue-fractured surfaces of baseline CFRP laminates. The fiber/matrix interfacial debonding was observed after the fatigue tests, reducing the post-impact fatigue strength remarkedly. The SEM images shown in Figure 15b,c verify the reinforcement mechanisms of MWCNTs to improve the residual fatigue strength of the MWCNT-modified CFRP laminates. The pullout of MWCNTs from the matrix and the bridging effect of MWCNTs delay the damage growth rate of matrix cracks significantly.

**Figure 16:**
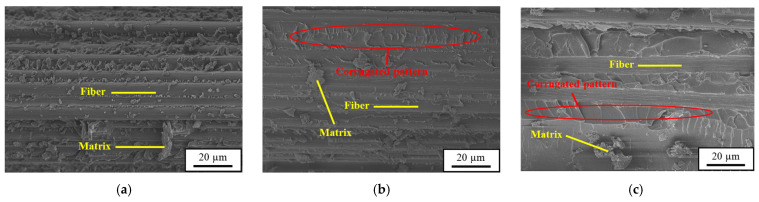
SEM images obtained after the post-impact fatigue tests for the (**a**) baseline CFRP specimen, (**b**) 0.3 wt.% MWCNT modified CFRP laminate specimen, and (**c**) 0.3 wt.% GNP modified CFRP laminate specimen.

Figure 16 shows the effect of applied nanoparticles in the matrix on the SEM images of the post-impact fatigue-fractured surfaces for the CFRP laminate specimens. Comparison between these fracture surfaces indicates that more matrix debris attached on the fracture surface is observed for the nano-modified specimens than the ones with a neat epoxy matrix. The application of nanoparticles provides crosslinks between fiber/matrix interfaces, further delays the development of fiber/matrix debonding and delamination growth. Furthermore, the nano-modified CFRP laminates present more obvious corrugated fracture surfaces than the baseline CFRP laminates. These wavy striations provide evidence that the matrix cracks change the direction of propagation to bypass the encountering nanoparticles. The so-called crack deflection effect caused by nanoparticles further improves the fatigue resistance of the impacted CFRP laminates for more energy needs to be dissipated when the matrix cracks bypass these nanoparticles. Furthermore, the deflection effect resulting from GNPs (Figure 16c) is expected to be more significant than that caused by MWCNTs (Figure 15c and Figure 16c) for the aspect ratio of layer-shaped GNPs are larger than that of MWCNTs.

## 4. Conclusions

The experimental results of the impact force history and force-deflection curves obtained in the impact tests demonstrate that mixing MWCNTs and GNPs in the matrix can improve the impact resistance of CFRP laminates and reduce the impact damage area and dent depth effectively. Furthermore, the post-impact tensile static strength and fatigue life of the CFRP laminates can be improved by reducing the impact damage. The improvement in the impact resistance and post-impact tensile static and fatigue strengths is found to increase with the applied nanoparticle concentrations. This study employed infrared thermal images obtained at fixed cycle numbers to monitor the evolution of the damage area during the fatigue test. A linear model was applied to describe the relationship between the damage area and fatigue cycles successfully, and the damage area growth rate can be obtained from the fitting data. The damage area growth rates of the nano-modified CFRP laminates observed in the post-impact fatigue tests are much lower than those of the baseline CFRP laminates. Similarly, the specimens with the matrix modified by more nanoparticles display stronger resistance to fatigue damage propagation. However, the agglomeration of nanoparticles caused by the overdose of GNPs is unhelpful to retard the fatigue damage evolution rate. This study also demonstrates that the damage growth rates between MWCNT-modified laminates and GNP-modified laminates are slight; the MWCNT-modified laminates present longer post-impact fatigue lives because smaller as-impact damage is initiated after the impact test. The pullout effect of MWCNTs and crack deflection effect caused by the applied carbon nanofillers were the main mechanisms to enhance the impact resistance and the post-impact strength of the CFRP laminates.

## Figures and Tables

**Table 1:** Experimental results of maximum impact force, absorbed energy, damage area, dent depth and post-impact statically tensile strength for the studied nano-modified CFRP laminate specimens with various configuration.

Nanoparticle Type	Nanoparticle Concentration (wt.%)	Maximum Impact Force (N)	Absorbed Energy (J)	Damage Area (mm^2^)	Dent Depth (N)	Tensile Strength After Impact *σ*_TAI_ (MPa)
Neat epoxy	0	1486.33 ± 57.63	11.77 ± 0.18	177.00 ± 43.82	1.04 ± 0.089	214.55 ± 0.54
MWCNTs	0.1	1570.66 ± 85.76	11.80 ± 0.28	161.33 ± 1.62	0.72 ± 0.091	225.31 ± 1.94
	0.3	1710.00 ± 83.14	11.70 ± 0.03	130.00 ± 16.77	0.69 ± 0.046	242.32 ± 1.40
	0.5	1874.66 ± 86.26	11.55 ± 0.24	129.00 ± 8.83	0.68 ± 0.057	259.18 ± 3.79
GNPs	0.1	1675.00 ± 53.88	11.68 ± 0.06	163.00 ± 12.24	0.84 ± 0.081	220.44 ± 0.25
	0.3	1717.33 ± 99.60	11.64 ± 0.03	142.33 ± 16.99	0.83 ± 0.012	228.00 ± 1.48
	0.5	1721.66 ± 119.33	12.20 ± 0.21	120.67 ± 7.48	0.81 ± 0.030	231.65 ± 7.95

**Table 2:** Experimental results of post-impact fatigue life for the studied nano-modified CFRP laminate specimens with various configurations.

Nanoparticle Type	Nanoparticle Concentration (wt.%)	Load Level (%)	Fatigue Life *N_f_* (Cycles)	Fatigue Life Average and Standard Deviation (Cycles)
Neat epoxy	0	80	>90,000, >90,000, >90,000	-
		90	14,692, 15,198, 12,153	14,014 ± 1322
MWCNTs	0.1	80	>90,000, >90,000, >90,000	-
		90	34,698, 29,572, 27,809	30,693 ± 2921
	0.3	80	>90,000, >90,000, >90,000	-
		90	32,485, 30,127, 38,464	33,692 ± 3508
	0.5	80	>90,000, >90,000, >90,000	-
		90	32,203, 37,541, 36,566	35,436 ± 2320
GNPs	0.1	80	>90,000, >90,000, >90,000	-
		90	14,305, 24,648, 18,055	19,002 ± 4275
	0.3	80	>90,000, >90,000, >90,000	-
		90	25,945, 19,986, 21,089	22,340 ± 2588
	0.5	80	>90,000, >90,000, >90,000	-
		90	39,137, 41,199, 39,545	39,960 ± 891

**Table 3:** Fitting results of post-impact damage area growth rates for the studied nano-modified CFRP laminate specimens with various configurations.

Nanoparticle Type	Nanoparticle Concentration (wt.%)	Damage Area Growth Rate, *dA*/*dN* (10^−3^ mm^2^/Cycle)		Coefficient of Determination *R*-Squared	
		80% Load Level	90% Load Level	80% Load Level	90% Load Level
Neat epoxy	0	2.22	8.65	0.98	0.92
MWCNTs	0.1 0.3 0.5	2.01 1.87 1.69	6.12 5.68 4.54	0.86 0.83 0.93	0.92 0.92 0.89
GNPs	0.1 0.3 0.5	2.07 1.65 1.80	5.40 5.10 5.62	0.94 0.86 0.99	0.96 0.84 0.94

## Data Availability

The original contributions presented in this study are included in the article. Further inquiries can be directed to the corresponding author.
